# Patient acceptance and experience with contrast-enhanced mammography in women with a personal history of breast cancer: findings from the COMBO trial

**DOI:** 10.1186/s13244-026-02434-x

**Published:** 2026-10-16

**Authors:** Giulia Vatteroni, Riccardo Levi, Marco Filippini, Martina Costa, Rubina Manuela Trimboli, Daniela Bernardi

**Affiliations:** 1 Department of Biomedical Sciences, Humanitas University, Pieve Emanuele, Milan, Italy; 2 Radiology Department, IRCCS Humanitas Research Hospital, Rozzano, Milan, Italy; 3 Radiology Department, ASST Spedali Civili, Brescia, Italy; 4 Radiology department, Presidio Ospedaliero Vittorio Emanuele, Gela, Italy

**Keywords:** Breast cancer survivors, Contrast enhanced mammography (CEM), Digital mammography, Experience, Follow-up

## Abstract

**Objectives:**

To evaluate patient acceptance, tolerability and experience with contrast-enhanced mammography (CEM) in women with a personal history of breast cancer undergoing routine post-surgical surveillance.

**Materials and methods:**

This prospective cohort study, conducted within the COMBO Trial, included consecutive women with a personal history of breast cancer undergoing CEM instead of digital mammography (DM) for routine surveillance from January 2023 to February 2025. Immediately after the examination, participants completed an anonymous 16-question questionnaire assessing discomfort, pain, exam duration, pre-examination reassurance, information received, anxiety, contrast injection, satisfaction and comparative experience with prior DM. Responses used Likert-type scales. Descriptive and non-parametric analyses were performed (*p* < 0.05 statistically significant).

**Results:**

Of 969 eligible women, 936 (mean age 58.2 years, SD ± 10.2 years) completed the questionnaire. CEM was perceived as comparable to prior DM in terms of discomfort (631/936; 67.4%), pain (725/936; 77.4%), positioning (717/936; 76.6%) and duration (601/936; 64.3%). Overall satisfaction was high (900/936; 96.2%), and 932/936 (99.6%) of participants would recommend CEM. Although 433/936 (46.3%) of women reported moderate-to-high pre-examination anxiety, higher anxiety was significantly associated with greater perceived discomfort, pain, uncomfortable positioning, longer examination duration, lower reassurance, lower comfort during contrast medium administration, and lower overall satisfaction (all *p* < 0.001).

**Conclusions:**

In this large cohort of breast cancer survivors, CEM demonstrated high acceptance, satisfaction and tolerability in post-surgical follow-up. Although pre-examination anxiety negatively influenced examination experience, it did not reduce patients’ willingness to undergo CEM, supporting its integration into personalised post-treatment breast cancer surveillance.

**Key Points:**

***Question*** Evidence on patient acceptance and tolerability of CEM for routine post-surgical surveillance in women with previous breast cancer is limited.

***Findings*** In 936 breast cancer survivors, CEM showed high satisfaction and tolerability, while pre-examination anxiety was associated with a less favourable experience.

***Critical Relevance Statement*** High patient acceptance supports CEM as a feasible surveillance option after breast cancer treatment, while the association between anxiety and poorer experience highlights the radiologist’s role in pre-examination communication and reassurance.

**Graphical Abstract:**

**Figure:**
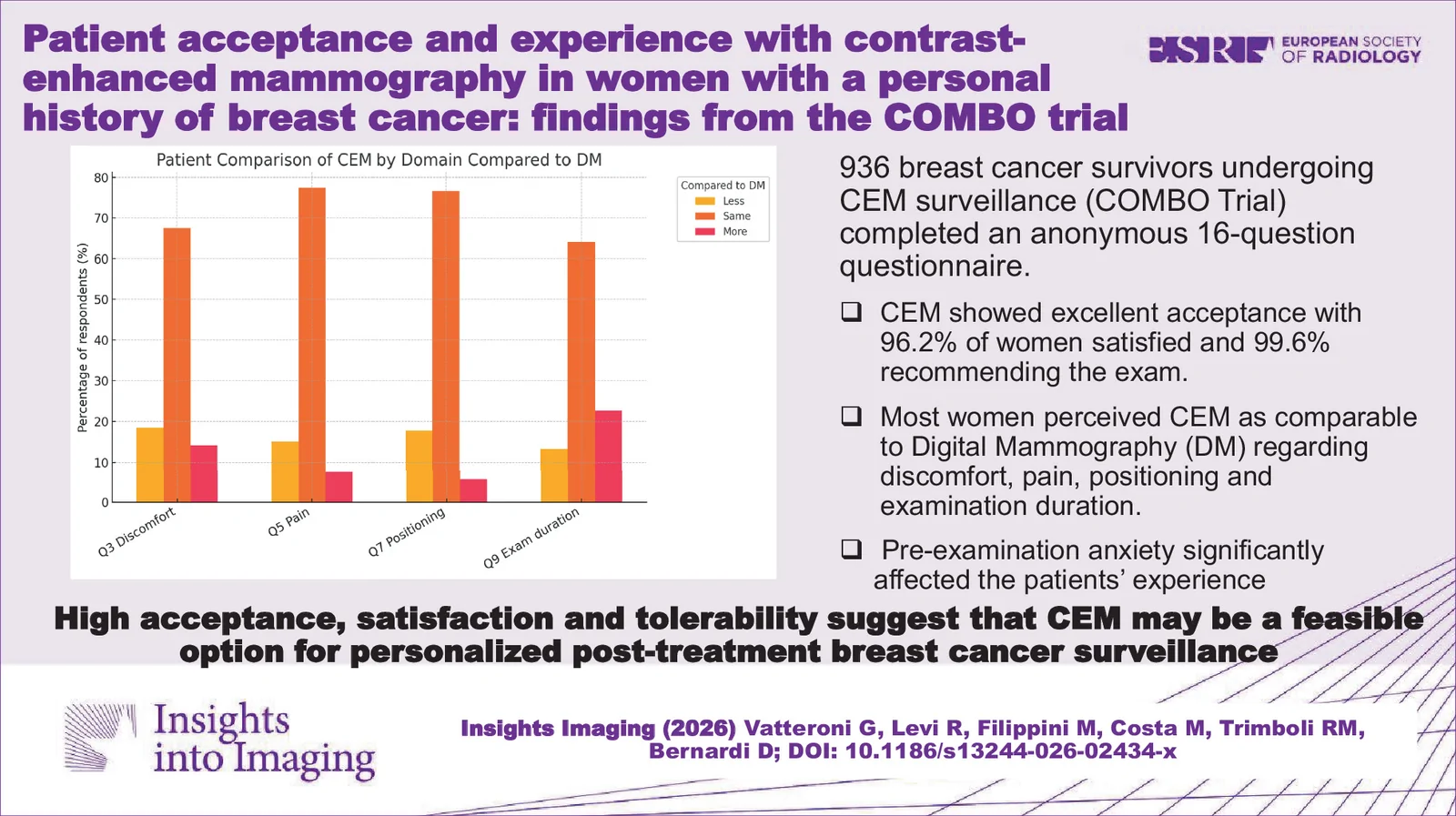

## Background

Women with a personal history of breast cancer (PHBC) are categorised as intermediate-risk patients (lifetime risk 15–20%) requiring long-term imaging surveillance due to an elevated risk of developing either local recurrence or new primary tumours. In this population, digital mammography (DM) remains the standard imaging modality for follow-up, however, its sensitivity is limited due to post-surgical changes [1]. These limitations may compromise early detection, especially in the first years following initial treatment. Therefore, additional or alternative imaging strategies may be beneficial in this group of patients in whom the diagnostic performance of DM may be limited. Contrast-enhanced mammography (CEM), is an imaging tool that provides both morphological and functional information through the use of iodinated contrast agents to highlight tumour-associated neovascularisation [2]. CEM has demonstrated diagnostic performance comparable to that of breast magnetic resonance imaging (MRI) in various clinical contexts, including preoperative staging and supplemental screening [3, 4], with increased cancer detection in post-surgical surveillance [5–7].

The European Society of Breast Imaging (EUSOBI) has recently released recommendations advocating annual DM for five years after surgery, with supplemental imaging using MRI or CEM in breast cancer survivors with specific risk factors, such as dense breast tissue or a diagnosis before the age of 50 years [8]. Despite its diagnostic advantages, CEM differs from DM in ways that may affect patient experience. Specifically, it requires intravenous (IV) contrast medium administration and involves slightly longer examination times and higher radiation exposure, which may influence patient tolerability and acceptance.

Although several studies have investigated patient preferences and experiences with CEM in various clinical contexts—including neoadjuvant treatment [9], preoperative staging [10], and supplemental screening [11–13], evidence specifically focusing on women undergoing follow-up after breast cancer surgery remains limited, as summarised in Table 1. In this last subgroup, existing studies are based on small cohorts [14, 15], hypothetical preference modelling [16] or evaluations of patient attitudes during appointments where CEM was not actually performed [11]. Patient acceptability is a key determinant of adherence to follow-up protocols and a well-tolerated imaging modality may significantly improve long-term outcomes by ensuring consistent participation in surveillance programs.

**Table 1:** Summary of published studies evaluating patient experience, preference, and tolerability of CEM across different clinical settings

Study	No. of patients	Clinical setting	Survey timing	Survey administration method	Comparison type	Main study conclusion
Hobbs et al [10]	49	Preoperative staging (biopsy-proven breast cancer)	After both CEM and MRI	Paper-based Likert scale questionnaire	Direct comparison CEM vs MRI	Patients preferred CEM vs MRI: less noise, greater comfort, shorter duration, less anxiety
Phillips et al [18]	118	High-risk screening	Post-imaging	Paper-based Likert-style questionnaire	Direct preference CEM vs MRI	More patients preferred CEM over MRI; better comfort reported
Miller et al [12]	508	Intermediate-risk screening (dense breast)	During screening mammography (Mar 2017–Feb 2018)	Tablet-based survey in waiting room	Hypothetical preference and deterrents	Patients preferred higher sensitivity even with IV contrast or higher cost
Son et al [11]	1011	General screening population	During mammography appointment (Dec 2019–Mar 2020)	Paper-based survey completed anytime during visit	Hypothetical preference: CEM vs MRI	Many women favoured sensitive exams like CEM or ABMRI
Patel et al [14]	410	Intermediate- to high-risk women (including 98 PHBC women)	Within 2 weeks after CEM	Test morbidity index (TMI) survey via email	Tolerability and acceptability of CEM only	CEM was well tolerated; most women would repeat it
Moravia et al [15]	104	Intermediate- risk women (PHBC women)	Up to 1 month after CEM	Verbal survey in person or by phone, entered into REDCap	Annual CEM experience and preference compared to MRI and DM	CEM was acceptable and preferred annually by most women with PHBC
Pires-Gonçalves et al [9]	38	Neoadjuvant chemotherapy (NAC) (CEM vs MRI repeated exams)	During NAC imaging sequence	Self-reported questionnaire (5-point Likert) after each imaging exam	Repeated CEM vs MRI during NAC (tolerability)	CEM was preferred in 76% of repeated NAC cases due to comfort and speed
Berg et al [16]	222	Supplemental screening (including 185 PHBC women)	Post-imaging (after both exams)	Survey via email. Online analytic hierarchy process (AHP) model	Post-imaging preference trade-off (AHP model): CEM vs MRI	CEM preferred over MRI in 71% of cases; positioning and anxiety were key factors
Miller et al [19]	163	Intermediate- risk women (dense breast)	Pre- and post-imaging	Tablet-based survey in clinic	CEM screening tolerability in dense breasts	CEM was well tolerated; 91% would repeat, 95% would recommend
Allajbeu et al [13]	159	intermediate risk screening (dense breast)	Immediately after the imaging examination, baseline or follow-up (18 months)	Mixed-methods approach, adapted from the Testing Morbidity Index using a tailored questionnaire, Likert scales and open-text responses	Patient acceptance of supplemental imaging (ABUS, CEM, AB-MRI) vs standard DM	Supplemental imaging is well accepted; clear communication reduces anxiety and improves experience

*AB-MRI* abbreviated breast magnetic resonance imaging, ABUS automated breast ultrasound, *NAC* neoadjuvant chemotherapy, TMI testing morbidity index

For this reason, understanding how this subset of patients experiences and accepts CEM is critical for evaluating its feasibility and its potential implementation in routine post-surgical surveillance.

To address this research gap, the present study aims to assess patient acceptance and experience of CEM compared to standard DM in women undergoing breast cancer follow-up imaging using an anonymous questionnaire.

## Material and methods

Survey data were collected as part of an institutional review board–approved prospective intra-individual comparison study, the COMBO Trial, which included women with a personal history of invasive breast cancer or ductal carcinoma in situ treated with breast-conserving surgery. From January 2023 to February 2025, participants were invited to undergo CEM for routine breast cancer follow-up, as their first surveillance CEM examination at Humanitas Research Hospital.

Informed consent was obtained from all participants. The target population was composed of asymptomatic women who underwent conservative surgery for breast cancer within the previous 10 years. Patients who did not meet the study inclusion criteria (women with breast symptoms suspicious for breast cancer, a previous history of allergy to iodinated contrast agents, renal impairment or breast implants) were excluded from the analysis. Patients were also excluded due to unsuccessful IV access placement or incomplete survey responses.

All CEMs were performed with a Mammomat Revelation system (Siemens Healthineers). A non-ionic low-osmolar iodinated contrast agent (Omnipaque 350 mg/mL, GE) was injected at a volume of 1.5 mL/kg body weight (maximum of 120 mL) at a rate of 2.5 mL/s using a power injector. After 2 min from the contrast injection, paired low-energy and high-energy images were acquired with the breast in compression for the four standard craniocaudal and mediolateral oblique views, starting from the operated breast. Additional delayed acquisitions of the operated breast (both craniocaudal and mediolateral oblique) were performed after 6 min from the contrast injection. Recombined images for each acquired projection were generated using post-processing.

Immediately after the examination, an anonymous 16-question questionnaire was administered to the patient. Each participant underwent a single CEM examination at study entry and contributed only one questionnaire to the present analysis; therefore, no repeated observations from the same patient were included.

The questionnaire was specifically developed for the COMBO Trial by the study investigators, all breast radiologists, based on domains previously explored in studies investigating patient experience, preference and tolerability of CEM. Patients, psychologists, or other healthcare professionals were not involved in questionnaire development, and no pilot testing was performed before study initiation.

The questionnaire covered several domains, including perceived discomfort, pain, positioning, exam duration, pre-examination reassurance, adequacy of information provided, anxiety related to contrast medium administration, acceptance of risk of a contrast reaction if it helps detect cancer earlier, and overall satisfaction with CEM. The questionnaire was administered immediately after CEM, before the examination results were available to the patients. The term reassurance referred specifically to the extent to which patients felt reassured by the radiologist before undergoing CEM, particularly regarding any concerns related to the examination and was therefore independent of the diagnostic outcome. The questionnaire also compared the current CEM examination with patients’ recalled experience of their previous surveillance DM, generally performed approximately 12 months earlier; thus, this represented a retrospective patient-reported comparison rather than a contemporaneous head-to-head assessment of the two modalities.

The full questionnaire is provided in Table 2.

**Table 2:** Summary of patient responses to the post-examination CEM questionnaire

*N*	(%)
Q1. Was the exam interrupted?	
No	936 (100%)
Yes	0
Q2. How much discomfort did you experience during the exam?	
5—Very severe discomfort	10 (1.1%)
4—Severe discomfort	60 (6.4%)
3—Moderate discomfort	182 (19.4%)
2—Mild discomfort	339 (36.2%)
1—No discomfort	345 (36.9%)
Q3. Compared to your usual mammogram, how would you rate the discomfort of this exam?	
5—Much more discomfort	11 (1.2%)
4—More discomfort	121 (12.9%)
3—Same level of discomfort	631 (67.4%)
2—Less discomfort	100 (10.7%)
1—Much less discomfort	73 (7.8%)
Q4. How much pain did you experience during the exam?	
5—Very severe pain	23 (2.5%)
4—Severe pain	71 (7.6%)
3—Moderate pain	223 (23.8%)
2—Mild pain	327 (34.9%)
1—No pain	292 (31.2%)
Q5. Compared to DM, how would you rate the pain from this exam?	
3—More painful	70 (7.5%)
2—Same level of pain	725 (77.4%)
1—Less painful	141 (15.1%)
Q6. How uncomfortable was the positioning during the exam?	
5—Very uncomfortable	2 (0.2%)
4—Uncomfortable	44 (4.7%)
3—Moderately uncomfortable	189 (20.2%)
2—Slightly uncomfortable	303 (32.4%)
1—Not uncomfortable at all	398 (42.5%)
Q7. Compared to DM, how would you rate the positioning?	
3—More uncomfortable	53 (5.7%)
2—Same level of uncomfortable positioning	717 (76.6%)
1—Less uncomfortable	166 (17.7%)
Q8. Did you find the exam long?	
5—Very long	1 (0.1%)
4—Fairly long	27 (2.9%)
3—Moderately long	205 (21.9%)
2—Slightly long	302 (32.3%)
1—Not long at all	401 (42.8%)
Q9. Compared to DM, how would you rate the duration of this exam?	
3—Longer	211 (22.5%)
2—Same duration	601 (64.3%)
1—Shorter	124 (13.2%)
Q10. To what extent did you feel reassured by the radiologist before undergoing the examination?	
5—Completely reassured	667 (71.3%)
4—Mostly reassured	199 (21.3%)
3—Moderately reassured	62 (6.6%)
2—Slightly reassured	4 (0.4%)
1—Not reassured at all	4 (0.4%)
Q11. How well informed did you feel about the exam?	
5—Very well informed	703 (75.1%)
4—Well informed	186 (19.9%)
3—Moderately informed	39 (4.2%)
2—Poorly informed	3 (0.3%)
1—Not informed at all	5 (0.5%)
Q12. How much anxiety did the CEM cause you?	
5—Very high anxiety	54 (5.8%)
4—High anxiety	144 (15.4%)
3—Moderate anxiety	235 (25.1%)
2—Low anxiety	328 (35.0%)
1—No anxiety	175 (18.7%)
Q13. How well did you tolerate the administration of the contrast agent?	
5—Very well	305 (32.6%)
4—Fairly well	417 (44.6%)
3—Moderately well	150 (16.0%)
2—Poorly	49 (5.2%)
1—Very poorly	15 (1.6%)
Q14. Would you accept the small risk of a contrast reaction if it helps detect cancer earlier?	
5—Definitely yes	644 (68.8%)
4—Probably yes	204 (21.8%)
3—Possibly	67 (7.2%)
2—Probably not	15 (1.6%)
1—Definitely not	6 (0.6%)
Q15. Overall, how satisfied are you with the CEM?	
5—Very satisfied	660 (70.5%)
4—Satisfied	240 (25.6%)
3—Neutral	24 (2.6%)
2—Dissatisfied	3 (0.3%)
1—Very dissatisfied	9 (0.9%)
Q16. Would you recommend this exam to others?	
Yes	932 (99.6%)
No	4 (0.4%)

Questions 2, 3, 4, 6, 8, 10, 11, 12, 13, 14, and 15 were rated using a 5-point Likert scale (1 = ‘Strongly disagree’ to 5 = ‘Strongly agree’). Question 1 had a binary response format, while questions 5, 7, and 9 were assessed using a 3-point Likert scale. The final question (16) included a binary response domain asking whether the patient would recommend CEM, followed by an open-ended prompt inviting participants to explain the reasons for their response.

All questionnaire responses were entered into a dedicated electronic database by the research coordinator.

### Statistical analysis

Descriptive statistics were used to summarise patient responses to each question. For categorical variables, frequencies and percentages were reported. For questions assessed using 3- or 5-point Likert scales, response distributions, means, and standard deviations (SD) were calculated for descriptive purposes.

To investigate the relationships among patient-reported outcomes, pairwise associations between questions were evaluated using Spearman’s rank correlation coefficient, accounting for the ordinal nature of the data. Correlation analyses focused on the associations between pre-examination anxiety, perceived examination experience (discomfort, pain, positioning, examination duration), reassurance, adequacy of pre-examination information, comfort during contrast medium administration, willingness to accept the small risk of contrast-related adverse events, and overall satisfaction. The association between age and patient-reported measures of CEM experience and acceptance was also assessed using Spearman’s rank correlation, with age analysed as a continuous variable.

An exploratory subgroup analysis was subsequently performed to identify factors associated with the perception of pain during CEM compared with previous DM. Women who reported CEM as less painful than DM were compared with those reporting CEM as more painful than DM. Patients reporting no difference between the two examinations were excluded to allow comparison between the two opposite patient perceptions. No a priori power calculation was performed for this exploratory subgroup analysis. Differences between groups were assessed using the Mann–Whitney U test for ordinal variables, and the χ² test or Fisher’s exact test, as appropriate, for categorical variables.

A *p*-value < 0.05 was considered statistically significant. All analyses were performed using the SciPy library, Python language (version 3.11).

## Results

Of the 969 women initially enroled in this prospective study, 5 were excluded since they did not meet the inclusion criteria, and 28 were classified as early terminations due to IV access failure. Consequently, 936 patients (mean age 58.2 years, SD ± 10.2 years) successfully underwent CEM and all of them completed the questionnaire; thus, they were included in the final analysis.

Mean discomfort during CEM was low (1.99 ± 0.96), with only 70/936 (7.5%) reporting high discomfort. When compared with prior DM, 631/936 (67.4%) perceived similar discomfort and 132/936 (14.1%) reported greater discomfort with CEM.

Mean pain score was 2.15 ± 1.03, with 619/936 (66.1%) reporting mild or no pain and 94/936 (10.0%) reporting severe pain. Compared with previous DM, only 70/936 (7.5%) perceived CEM as more painful.

Breast positioning was rated as not or only slightly uncomfortable by 701/936 (74.9%) of participants (mean 1.88 ± 0.91). In comparison with prior DM, 717/936 (76.6%) perceived similar positioning discomfort.

Examination duration was perceived as not long by 401/936 (42.8%) of women (mean 1.85 ± 0.87), and 601/936 (64.3%) considered CEM duration comparable to DM.

**Fig. 1:**
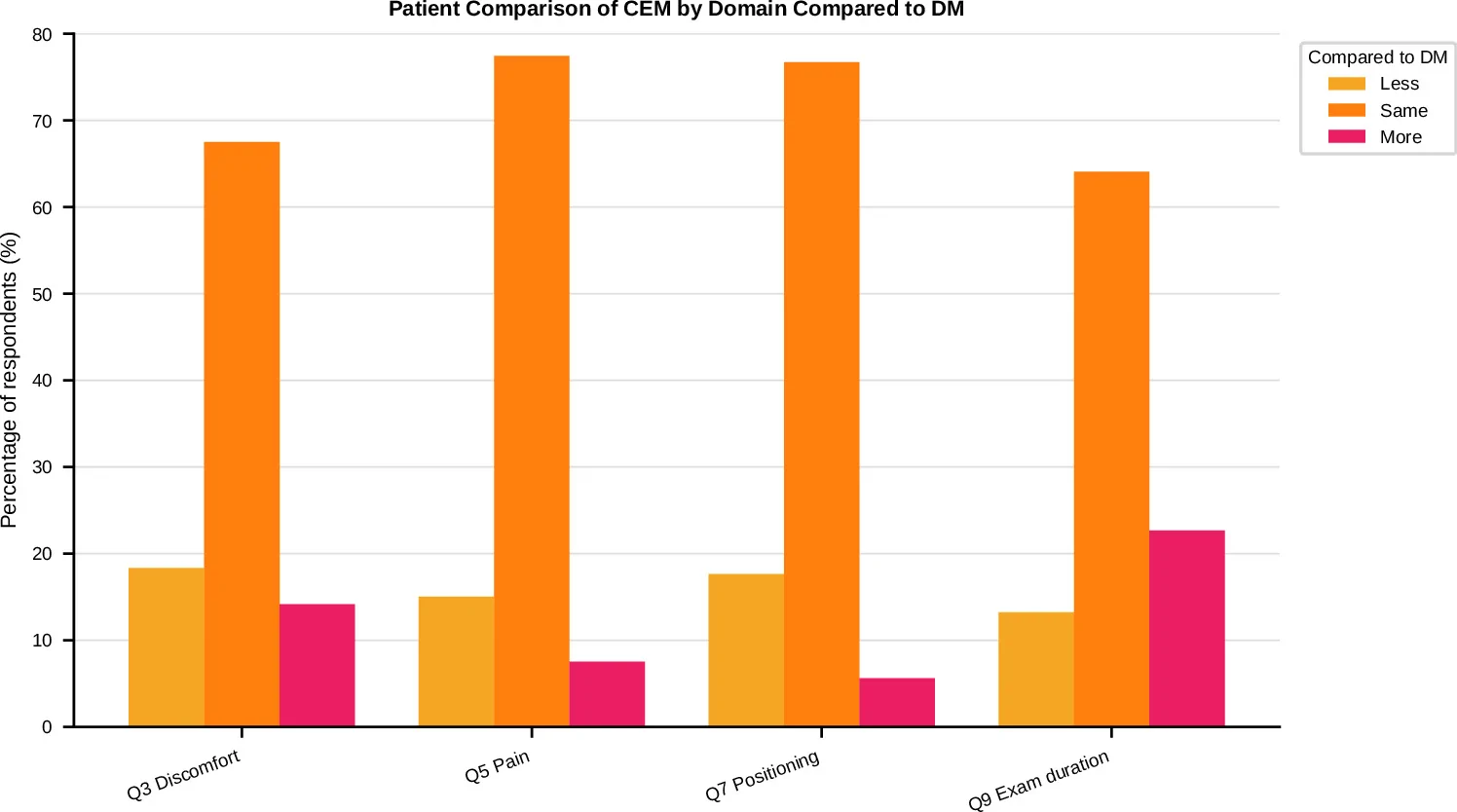
Patient-reported comparison of CEM and DM across four domains: discomfort, pain, positioning, and exam duration. Responses were grouped into three categories: “Less,” “Same,” or “More” compared to prior DM experience

Figure 1 summarizes patient-reported comparison of CEM and previous DM across four domains: discomfort, pain, positioning and exam duration.

High levels of reassurance (mean 4.63 ± 0.67) and perceived adequacy of pre-examination information (mean 4.69 ± 0.62) were reported, with over 90% assigning ratings ≥ 4 for both questions.

Nearly half of participants reported moderate-to-high anxiety related to contrast medium administration. The mean anxiety score was 2.54 (SD = 1.13): 503/936 (53.7%) patients reported no or low anxiety, 235/936 (25.1%) reported moderate anxiety, and 198/936 (21.2%) reported high or very high anxiety.

Despite this, IV contrast medium administration was generally well tolerated. The mean comfort score was 4.01 (SD = 0.92), with 722/936 (77.2%) women reporting high levels of comfort, 150/936 (16.0%) moderate comfort, and 64/936 (6.8%) poor or very poor tolerability of contrast medium administration.

The willingness to accept a minimal risk of contrast-related adverse events for potential earlier cancer detection was also very high, with a mean score of 4.57 ± 0.75 and 848/936 (90.6%) women rating this question with a 4 or 5, and only 21/936 (2.2%) not accepting the risk of contrast medium adverse reaction even if allows early detection.

Finally, overall satisfaction with the CEM examination was excellent. The mean score was 4.64 ± 0.64, with 900/936 (96.2%) of patients assigning a rating of 4 or 5.

The complete rating for all the questions is shown in Table 2.

**Fig. 2:**
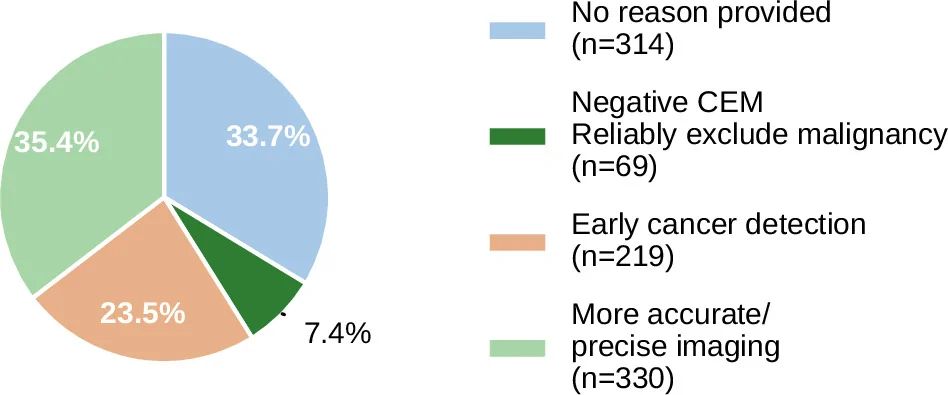
Reasons for recommending CEM among 932 women

Among the women willing to recommend CEM (932 out of 936), 314/932 (33.7%) did not provide a response to the open-ended question. Among women recommending CEM, the most frequently cited reasons were perceived higher diagnostic accuracy for 330/618 (53.4%) and earlier cancer detection for 219/618 (35.4%) (Fig. 2).

Conversely, 4/936 (0.4%) patients reported that they would not recommend CEM. Of these, only 1 (0.1%) provided a reason, considering CEM too invasive for routine screening and suggesting that its use should be reserved for specific cases. The remaining 3 (0.3%) did not answer the open-ended question.

Correlation analysis demonstrated that pre-examination anxiety was consistently associated with a less favourable examination experience. Higher anxiety scores were positively correlated with greater perceived discomfort (ρ = 0.372, *p* < 0.001), pain (ρ = 0.298, *p* < 0.001), positioning discomfort (ρ = 0.358, *p* < 0.001), and longer perceived examination duration (ρ = 0.367, *p* < 0.001). Conversely, anxiety was inversely correlated with reassurance (ρ = −0.247, *p* < 0.001), perceived adequacy of information (ρ = −0.190, *p* < 0.001), comfort during contrast medium administration (ρ = −0.260, *p* < 0.001), overall satisfaction (ρ = –0.225, *p* < 0.001), and willingness to accept the small risk of contrast-related adverse events for earlier cancer detection (ρ = –0.148, *p* < 0.001). Greater reassurance was positively associated with both overall satisfaction (ρ = 0.364, *p* < 0.001) and willingness to accept the small risk of contrast-related adverse events (ρ = 0.253, *p* < 0.001), while better perceived information was positively associated with overall satisfaction (ρ = 0.369, *p* < 0.001) and willingness to accept the small risk of contrast-related adverse events (ρ = 0.322, *p* < 0.001). The complete correlation matrix is provided in Supplementary Table S1.

Age showed statistically significant but negligible correlations with discomfort during CEM (ρ = −0.119, *p* < 0.001), pain during CEM (ρ = −0.107, *p* = 0.001), pre-examination reassurance (ρ = −0.088, *p* = 0.007), adequacy of information received (ρ = −0.066, *p* = 0.045), acceptance of contrast-related risk for earlier cancer detection (ρ = −0.083, *p* = 0.012), and overall satisfaction (ρ = −0.078, *p* = 0.017). No significant associations were observed with positioning discomfort (ρ = −0.031, *p* = 0.349), perceived examination duration (ρ = −0.004, *p* = 0.897), pre-examination anxiety (ρ = −0.007, *p* = 0.825), comfort during contrast medium administration (ρ = 0.014, *p* = 0.663), or willingness to recommend CEM (ρ = 0.054, *p* = 0.096). Overall, all associations were of negligible magnitude (absolute Spearman’s ρ ≤ 0.119), suggesting a minimal influence of age on patient-reported CEM experience and acceptance. Complete results are provided in Supplementary Table S2. Among the 211 women included in the pain-based subgroup analysis, 70 perceived CEM as more painful than previous DM, whereas 141 perceived it as less painful. Women reporting greater pain during CEM also reported greater overall discomfort, more uncomfortable breast positioning, longer perceived examination duration, higher pre-examination anxiety, lower reassurance, lower comfort during contrast medium administration (*p* = 0.002), and lower overall satisfaction (all other *p* < 0.001). In contrast, no significant differences were observed regarding the adequacy of pre-examination information (*p* = 0.292), willingness to recommend CEM (*p* = 0.55), acceptance of the small risk of contrast-related adverse events for earlier cancer detection (*p* = 0.994), or examination interruption (*p* = 0.491).

## Discussion

CEM has been increasingly incorporated into risk-adapted imaging pathways; however, evidence supporting its use in post-treatment surveillance remains limited. Breast cancer survivors represent a clinically and psychologically unique population, characterised by a history of breast cancer, fear of recurrence, heightened risk perception and long-term follow-up requirements.

In this large prospective cohort of 936 consecutive women treated with breast-conserving surgery undergoing post-surgical breast cancer surveillance, CEM demonstrated high acceptance and tolerability within routine surveillance practice, with the majority of participants perceiving discomfort, pain, positioning, and examination duration as comparable to prior DM.

While overall satisfaction was high, pre-examination anxiety emerged as an important factor influencing patient experience. Pre-examination anxiety significantly influenced perceived pain, discomfort, and reassurance, highlighting the impact of psychological factors on the patient experience during CEM. This is particularly relevant in breast cancer survivors, who may approach follow-up imaging with increased vulnerability related to fear of recurrence.

Compared to the BRAID trial, which evaluated supplemental imaging modalities within a screening population of women with dense breasts [13, 17], our study focuses specifically on post-surgical surveillance. Unlike screening participants, breast cancer survivors undergo imaging within a framework of prior diagnosis and treatment, where reassurance, perceived diagnostic accuracy, and trust in the medical team may carry greater weight. Furthermore, the large size of our cohort compared with the BRAID CEM subgroup strengthens the assessment of patient tolerability in a real-world surveillance setting.

Our results are consistent with prior reports demonstrating high patient acceptance of CEM in different settings. Patel et al [14] described favourable testing burden scores in intermediate- to high-risk women undergoing supplemental screening, while Moravia et al [15] reported high willingness to repeat annual CEM among breast cancer survivors. However, these studies were limited by smaller cohorts or shorter follow-up intervals. In addition, our data provide real-world confirmation of the preference modelling reported by Berg et al [16], in which CEM was ranked highly among surveillance options based on perceived diagnostic value and tolerability, despite the absence of direct examination experience in that analysis.

From a practical perspective, IV contrast medium administration remains the main procedural distinction between CEM and DM. In our cohort, IV placement failure occurred in 28/969 (2.9%) patients, highlighting a manageable but relevant operational consideration for implementation. Among women who completed CEM, nearly half reported moderate to high anxiety related to contrast medium administration; nevertheless, more than 90% were willing to accept a small risk of adverse reaction in exchange for earlier cancer detection. These findings suggest that perceived diagnostic benefit outweighs procedural concerns in most breast cancer survivors.

Importantly, our data emphasise that effective pre-examination communication plays a central role in patient experience. Women with higher pre-examination anxiety reported lower reassurance and greater perceived discomfort, highlighting the potential role of targeted communication strategies in mitigating distress. These data are in line with the literature [15], suggesting that emotional and psychological factors can influence exam perception and underline the importance of pre-procedural counselling and effective communication to optimise the patient experience and acceptance. This underscores the pivotal role of the radiologist in the pre-examination phase, not only in communicating technical information, but in building trust and addressing anxiety. When women are adequately informed, the perceived diagnostic value of CEM outweighs concerns about discomfort or invasiveness [11]. Taken together, these findings suggest that CEM may represent a well-tolerated and patient-accepted imaging option within personalised post-treatment surveillance pathways, particularly in women for whom DM has limitations.

Nonetheless, several limitations must be acknowledged. First, participants voluntarily underwent CEM within a research setting, introducing potential selection bias and potentially leading to an overestimation of CEM acceptance. Moreover, women who were unable to undergo CEM because of unsuccessful IV access placement did not complete the questionnaire, and their exclusion may have contributed to an overestimation of CEM tolerability. As patient-reported measures were collected only after CEM completion, direct comparison with these women was not possible.

Second, the comparison between CEM and DM was based on patients’ retrospective perception of a previous DM examination, usually performed 12 months earlier, rather than on a contemporaneous head-to-head assessment of the two modalities. This may have introduced recall bias, as memories of the prior examination may be inaccurate and the more recent CEM experience may disproportionately have influenced patients’ judgement.

In addition, our research lacks paired, contemporaneous data, which may limit the reliability of direct comparisons between modalities. Furthermore, the pain-based subgroup analysis was exploratory and involved relatively small and unequal groups. As no a priori power calculation was performed for this analysis, limited statistical power, particularly for detecting small between-group differences, cannot be excluded.

Third, the questionnaire used in this study, although specifically designed for this population, was not based on a previously validated instrument such as the Testing Morbidities Index, and no pilot testing was performed before study initiation. These methodological limitations should be considered when interpreting the findings and may limit their reproducibility and cross-study comparability. Finally, longitudinal evaluation of acceptance over repeated surveillance rounds was beyond the scope of the present analysis.

In conclusion, CEM demonstrated high patient acceptance and tolerability in women with a PHBC undergoing post-surgical surveillance, with an examination experience largely comparable to previous DM. Although pre-examination anxiety negatively influenced patients’ perception of the examination, it did not reduce their willingness to undergo CEM. Overall, the high levels of patient acceptance observed in this study may facilitate the implementation of CEM within personalised post-surgical surveillance pathways and underscore the central role of structured communication in optimising patient experience.

## Supplementary information

ELECTRONIC SUPPLEMENTARY MATERIAL

## Data Availability

Access to the data supporting this study may be granted upon reasonable request to the Principal Investigator (D.B.) or the study coordinator (G.V.) (giulia.vatteroni@gmail.com) and may require a data sharing agreement.

## Abbreviations

CEM: Contrast-enhanced mammography
DM: Digital mammography
IV: Intravenous
MRI: Magnetic resonance imaging
NAC: Neoadjuvant chemotherapy
PHBC: Personal history of breast cancer
SD: Standard deviation
