# Supplementary material for: Patient acceptance and experience with contrast-enhanced mammography in women with a personal history of breast cancer: findings from the COMBO trial
Source: Insights Imaging. 2026 Oct 16;17:262. doi: 10.1186/s13244-026-02434-x (PMC13675458; doi:10.1186/s13244-026-02434-x)
Supplement: Supplementary file 1 [file 13244_2026_2434_MOESM1_ESM.pdf]

Patient Acceptance and Experience with Contrast-Enhanced Mammography in Women with a Personal History of Breast Cancer: findings from the COMBO Trial

ELECTRONIC SUPPLEMENTARY MATERIAL

SUPPLEMENTARY TABLE S1

Pairwise Spearman correlation coefficients (ρ) and p values among patient-reported outcomes from the 16-item CEM questionnaire.

	Q2	Q3	Q4	Q6	Q8	Q10	Q11	Q12	Q13	Q14	Q15
Q2	ρ=1.000, p=0	ρ=0.416, p=<0.001	ρ=0.582, p=<0.001	ρ=0.496, p=<0.001	ρ=0.415, p=<0.001	ρ=-0.239, p=<0.001	ρ=-0.145, p=<0.001	ρ=0.372, p=<0.001	ρ=-0.161, p=<0.001	ρ=-0.049, p=0.132	ρ=-0.160, p=<0.001
Q3	ρ=0.416, p=<0.001	ρ=1.000, p=0	ρ=0.340, p=<0.001	ρ=0.315, p=<0.001	ρ=0.236, p=<0.001	ρ=-0.110, p=<0.001	ρ=-0.078, p=0.017	ρ=0.192, p=<0.001	ρ=-0.199, p=<0.001	ρ=-0.012, p=0.721	ρ=-0.160, p=<0.001
Q4	ρ=0.582, p=<0.001	ρ=0.340, p=<0.001	ρ=1.000, p=0	ρ=0.518, p=<0.001	ρ=0.386, p=<0.001	ρ=-0.160, p=<0.001	ρ=-0.104, p=0.001	ρ=0.298, p=<0.001	ρ=-0.102, p=0.002	ρ=-0.046, p=0.163	ρ=-0.099, p=0.002
Q6	ρ=0.496, p=<0.001	ρ=0.315, p=<0.001	ρ=0.518, p=<0.001	ρ=1.000, p=0	ρ=0.563, p=<0.001	ρ=-0.289, p=<0.001	ρ=-0.192, p=<0.001	ρ=0.358, p=<0.001	ρ=-0.177, p=<0.001	ρ=-0.095, p=0.003	ρ=-0.177, p=<0.001
Q8	ρ=0.415, p=<0.001	ρ=0.236, p=<0.001	ρ=0.386, p=<0.001	ρ=0.563, p=<0.001	ρ=1.000, p=0	ρ=-0.276, p=<0.001	ρ=-0.185, p=<0.001	ρ=0.367, p=<0.001	ρ=-0.169, p=<0.001	ρ=-0.120, p=<0.001	ρ=-0.156, p=<0.001
Q10	ρ=-0.239, p=<0.001	ρ=-0.110, p=<0.001	ρ=-0.160, p=<0.001	ρ=-0.289, p=<0.001	ρ=-0.276, p=<0.001	ρ=1.000, p=0	ρ=0.535, p=<0.001	ρ=-0.247, p=<0.001	ρ=0.218, p=<0.001	ρ=0.253, p=<0.001	ρ=0.364, p=<0.001
Q11	ρ=-0.145, p=<0.001	ρ=-0.078, p=0.017	ρ=-0.104, p=0.001	ρ=-0.192, p=<0.001	ρ=-0.185, p=<0.001	ρ=0.535, p=<0.001	ρ=1.000, p=0	ρ=-0.190, p=<0.001	ρ=0.177, p=<0.001	ρ=0.322, p=<0.001	ρ=0.369, p=<0.001
Q12	ρ=0.372, p=<0.001	ρ=0.192, p=<0.001	ρ=0.298, p=<0.001	ρ=0.358, p=<0.001	ρ=0.367, p=<0.001	ρ=-0.247, p=<0.001	ρ=-0.190, p=<0.001	ρ=1.000, p=0	ρ=-0.260, p=<0.001	ρ=-0.148, p=<0.001	ρ=-0.225, p=<0.001
Q13	ρ=-0.161, p=<0.001	ρ=-0.199, p=<0.001	ρ=-0.102, p=0.002	ρ=-0.177, p=<0.001	ρ=-0.169, p=<0.001	ρ=0.218, p=<0.001	ρ=0.177, p=<0.001	ρ=-0.260, p=<0.001	ρ=1.000, p=0	ρ=0.301, p=<0.001	ρ=0.314, p=<0.001
Q14	ρ=-0.049, p=0.132	ρ=-0.012, p=0.721	ρ=-0.046, p=0.163	ρ=-0.095, p=0.003	ρ=-0.120, p=<0.001	ρ=0.253, p=<0.001	ρ=0.322, p=<0.001	ρ=-0.148, p=<0.001	ρ=0.301, p=<0.001	ρ=1.000, p=0	ρ=0.389, p=<0.001
Q15	ρ=-0.160, p=<0.001	ρ=-0.160, p=<0.001	ρ=-0.099, p=0.002	ρ=-0.177, p=<0.001	ρ=-0.156, p=<0.001	ρ=0.364, p=<0.001	ρ=0.369, p=<0.001	ρ=-0.225, p=<0.001	ρ=0.314, p=<0.001	ρ=0.389, p=<0.001	ρ=1.000, p=0

SUPPLEMENTARY TABLE S2

Association between age and patient-reported CEM outcomes

Spearman rank correlations using age as a continuous variable

Question	Outcome	Scale	N	Spearman ρ	P value
Q2	Discomfort during CEM	5-point Likert	936	-0.119	<0.001
Q3	Discomfort compared with prior DM	3-point Likert	936	-0.082	0.012
Q4	Pain during CEM	5-point Likert	936	-0.107	0.001
Q5	Pain compared with prior DM	3-point Likert	936	-0.082	0.012
Q6	Positioning discomfort	5-point Likert	936	-0.031	0.349
Q7	Positioning discomfort compared with prior DM	3-point Likert	936	-0.056	0.090
Q8	Perceived examination duration	5-point Likert	936	-0.004	0.897
Q9	Examination duration compared with prior DM	3-point Likert	936	-0.035	0.286
Q10	Pre-examination reassurance	5-point Likert	936	-0.088	0.007
Q11	Adequacy of information received	5-point Likert	936	-0.066	0.045
Q12	Pre-examination anxiety	5-point Likert	936	-0.007	0.825
Q13	Comfort during contrast medium administration	5-point Likert	936	0.014	0.663
Q14	Acceptance of contrast-related risk for earlier cancer detection	5-point Likert	936	-0.083	0.012
Q15	Overall satisfaction with CEM	5-point Likert	936	-0.078	0.017
Q16	Willingness to recommend CEM	binary	936	0.054	0.096

Note: Two-sided asymptotic p values are shown; statistical significance was defined as $p < 0.05$. Questionnaire items are identified according to the questionnaire in the main manuscript.
